# Association between blood pressure and Alzheimer disease measured up to 27 years prior to diagnosis: the HUNT Study

**DOI:** 10.1186/s13195-017-0262-x

**Published:** 2017-05-31

**Authors:** Jessica Mira Gabin, Kristian Tambs, Ingvild Saltvedt, Erik Sund, Jostein Holmen

**Affiliations:** 1 HUNT Research Centre, Faculty of Medicine and Health Sciences , Department of Public Health and Nursing, Norwegian University of Science and Technology (NTNU), Forskningsveien 2, 7600 Levanger, Norway; 2 Division of Mental Health, Norwegian Institute of Public Health, Post Office Box 4404, Nydalen, 0403 Oslo, Norway; 3 Faculty of Medicine and Health Sciences, Department of Neuromedicine and Movement Science, Norwegian University of Science and Technology (NTNU), Post Office Box 8905, 7491 Trondheim, Norway; 4 Department of Geriatrics, St. Olav’s Hospital, Post Office Box 3250, 7006 Trondheim, Norway

**Keywords:** Risk factors, Alzheimer disease, Vascular dementia, Blood pressure, Epidemiology, Prospective case cohort

## Abstract

**Background:**

A lot of attention has been paid to the relationship of blood pressure and dementia because epidemiological research has reported conflicting evidence. Observational data has shown that midlife hypertension is a risk factor for cognitive decline and dementia later in life, whereas there is evidence that low blood pressure is predictive in later life. The aim of the present study was to examine the association between dementia and blood pressure measured up to 27 years (mean 17.6 years) prior to ascertainment.

**Methods:**

In Nord-Trøndelag County, Norway, incident dementia data were collected during 1995–2011, and the diagnoses were validated by a panel of experts in the field. By using the subjects’ personal identification numbers, the dementia data were linked to data from the Nord-Trøndelag Health Study (the HUNT Study), a large, population-based health study performed in 1984–1986 (HUNT 1) and 1995–1997 (HUNT 2). A total of 24,638 participants of the HUNT Study were included in the present study, 579 of whom were diagnosed with Alzheimer disease, mixed Alzheimer/vascular dementia, or vascular dementia. Multiple logistic regression analyses were conducted to analyze the association between dementia and blood pressure data from HUNT 1 and HUNT 2.

**Results:**

Over the age of 60 years, consistent inverse associations were observed between systolic blood pressure and all-cause dementia, mixed Alzheimer/vascular dementia, and Alzheimer disease, but not with vascular dementia, when adjusting for age, sex, education, and other relevant covariates. This was observed for systolic blood pressure in both HUNT 1 and HUNT 2, regardless of antihypertensive medication use. There was an adverse association between systolic blood pressure, pulse pressure, and Alzheimer disease in individuals treated with antihypertensive medication under the age of 60 years.

**Conclusions:**

Our data are in line with those in previous studies demonstrating an inverse association between dementia and systolic blood pressure in individuals over the age of 60 years. We cannot exclude a survival effect, however. Among middle-aged subjects (<60 years), elevated systolic blood pressure and pulse pressure were associated with eventual Alzheimer disease in individuals who reported using antihypertensive medication.

**Electronic supplementary material:**

The online version of this article (doi:10.1186/s13195-017-0262-x) contains supplementary material, which is available to authorized users.

## Background

Blood pressure (BP) level is a commonly investigated vascular risk factor. Results reported to date in the epidemiological literature on BP and dementia are conflicting and unclear because researchers have faced challenges when examining individuals with declining cognition over time that impact findings [1]. Cross-sectional studies have shown both positive and negative correlations between hypertension and cognition. Longitudinal studies of the association of midlife BP and later AD have found that elevated BP predicts the development of Alzheimer disease (AD) [2–4]. Other longitudinal studies have suggested a J-shaped relationship whereby only a very high BP increases the risk of AD, and some studies have demonstrated a nonlinear association whereby both high and low BP are associated with cognitive decline or dementia [5–9]. Systolic blood pressure (SBP) variability has been associated with dementia, and greater variability was a predictor of faster disease progression in AD [10]. There have been studies in which researchers have found BP decline in patients with prevalent dementia, and few prospective studies have demonstrated that this decline is present prior to dementia onset [11, 12]. However, previous prospective studies on BP and dementia reported limitations due to small sample sizes, short durations of study follow-up, and limited data evaluating an age- and sex-dependent association [13, 14]. The aim of the present study was therefore to prospectively examine the relationship between BP measured up to 27 years prior to dementia diagnosis on the development of all-cause dementia, AD, vascular dementia (VaD), and a mixture of these in a population-based sample.

## Methods

### Setting and study population

Nord-Trøndelag County is located in central Norway and is a mostly rural and sparsely populated area. The geographic region covers 22,414 km^2^, which is comparable to the size of Wales. During the study period of 1995–2011, the largest of six small towns had a population of 21,000. According to census data of 2014, the population of Nord-Trøndelag County was 135,000 and stable, with a net outmigration of 0.3% per year. The population is predominantly of Caucasian ethnicity. There are 32 nursing homes in the county, as well as two regional hospitals, located in Namsos and Levanger.

#### HUNT study

The Helse Undersøkelse Nord-Trøndelag (1984–1986; HUNT 1 Study) was initiated in 1984–1986 as a health survey that addressed four main topics: hypertension, diabetes, quality of life, and tuberculosis as well as other lung diseases. The subsequent survey, Helse Undersøkelse Nord-Trøndelag (1995–1997; HUNT 2 Study) expanded the scientific program substantially and collected a large number of health-related data, as described in detail previously [15–17]. Briefly, all residents of Nord-Trøndelag County over the age of 19 years (with no upper age limit) were invited to participate. Postal invitations with an enclosed questionnaire asked participants to disclose information about their general health and were mailed to their home addresses prior to the clinical examination. A second questionnaire (Q2) asked participants to report on smoking and education status, alcohol consumption, and exercise habits. Q2 was distributed to participants who attended screening stations at their respective municipalities and was completed and returned by mail in a prepaid postal envelope. In total, 63,924 participants completed both questionnaires in HUNT 1, and 55,376 responded to both questionnaires in HUNT 2. All HUNT data are linked to the 11-digit personal identification number given to each Norwegian citizen at birth, enabling linkage to other health registers, such as the Health and Memory Study of Nord-Trøndelag.

#### Health and Memory Study of Nord-Trøndelag

The Health and Memory Study of Nord-Trøndelag (the HMS Study) had an aim to establish a database suitable as a basis for a large number of studies of dementia, and extensive information on the ascertainment process, measurements, and findings have been published previously [18]. Briefly, recruitment of patients diagnosed with dementia is formed from two panels. Panel 1 consisted of patients diagnosed with dementia at the two hospitals in the county in the period from 1995 to 2010. Specialists in geriatric medicine and geriatric psychiatry were responsible for the diagnostic workup of dementia at the two memory clinics. Dementia ascertainment is based on algorithms using the criteria set by the World Health Organization’s International Classification of Diseases, Tenth Revision (ICD-10). ICD-10 has been shown to be congruent with the *Diagnostic and Statistical Manual of Mental Disorders, Fourth Edition*, which sets criteria standards according to clinical examination, patient and caregiver history, blood samples, and imaging of the brain [19, 20]. Initiated by the HMS Study in 2010, the dementia diagnoses were validated by a panel of four specialists who reviewed electronic hospital records retrospectively and confirmed the presence of dementia, classified the dementia by type, and determined the year of onset. Panel 2 of the HMS Study recruited patients from all nursing homes in Nord-Trøndelag County. Initiated in June 2010, each nursing home resident received an individually written invitation to participate in the study. Thereafter, a team of nine specially trained registered nurses visited every nursing home and conducted clinical examinations and standardized interviews with patients, their closest caregivers, and their closest relatives. Time of onset was determined by asking next of kin or caregivers to recall the number of months the resident had displayed symptoms. Nursing home assessments were completed in March 2011, which marked the end of the study data collection. The dementia diagnosis was thereafter validated by a panel of two specialists who confirmed the presence of dementia and classified the dementia by subtype.

#### HMS participants

**Fig. 1:**
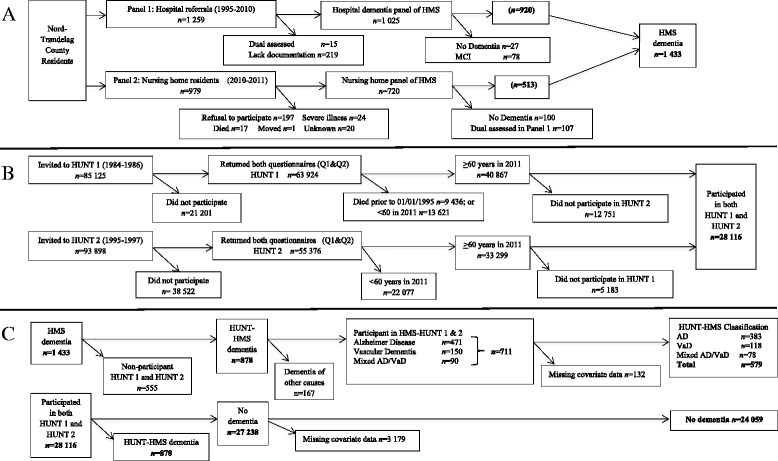
Flowchart of the present study describing the selection criteria for the HMS dementia panels (**a**); HUNT 1 and HUNT 2 (**b**); and how these two studies were linked (**c**). *HMS* Health and Memory Study of Nord-Trøndelag (1995–2010), *MCI* Mild cognitive impairment, *HUNT 1* Helse Undersøkelse Nord-Trøndelag (1984–1986), *HUNT 2* Helse Undersøkelse Nord-Trøndelag (1995–1997), *Q1* Questionnaire 1, *Q2* Questionnaire 2, *AD* Alzheimer disease, *VaD* Vascular dementia

Panel 1 consists of a total of 1259 patients referred to the hospital for suspicion of cognitive decline, and 15 patients were excluded because of dual registration, 219 lacked journal documentation, 27 had no dementia present, and 78 were diagnosed with mild cognitive impairment. Thus, a total of 920 patients were included in panel 1. Of 979 patients initially identified in panel 2, 197 refused to participate, 24 patients were excluded because of severe illness, 17 died, 1 moved, and 20 were excluded for unknown reasons. Additionally, 100 patients were excluded because they had no dementia, and 107 were excluded for dual assessment. Ultimately, 513 patients were included in panel 2, comprising of a total of 1433 patients in the HMS Study (Fig. 1a).

#### HUNT-HMS participants

In the present study, we examined data using selection criteria based on HUNT participants who were alive after 1 January 1995 and were over the age of 60 years in 2011. A total of 40,867 subjects from HUNT 1 and 33,299 from HUNT 2 fulfilled selection requirements. We examined BP data from both HUNT 1 and HUNT 2, and a total of 28,116 participated in both HUNT 1 and HUNT 2 (Fig. 1b). The national personal identification number was used to link the HMS Study with the HUNT data material. Of the 1433 diagnosed with dementia in the HMS Study, 878 participated in both HUNT 1 and HUNT 2. Of these, 167 were diagnosed with dementia of other causes and were excluded from the present study; 711 were diagnosed with AD, VaD, or mixed AD/VaD; and 579 had complete covariate data. The HUNT 1 and HUNT 2 participants who were not diagnosed with dementia constituted the remaining portion of the sample (*n* = 27,238), and 24,059 participants had complete covariate data (Fig. 1c).

### Baseline data

HUNT 1 stations featured standardized BP measurements for all participants and were managed by trained nurses or technicians using a mercury sphygmomanometer. The BP measurement routinely recorded readings with the participant in a sitting position after having rested a minimum of 5 minutes. SBP and diastolic blood pressure (DBP) were measured two times, and the second reading was used for our analyses. Heart rate, height, and weight were measured in HUNT 1 following a previously described protocol [16]. Sociodemographic data (age, sex, and education) were collected for the HUNT study following standardized protocols [17]. Covariates that are known to confound or mediate the association of dementia and BP were included in full model analyses. Education status, alcohol use, physical activity, subjective general health, cardiovascular disease, body mass index, waist-to-hip ratio, smoking status, antihypertensive tablet use, serum cholesterol, nonfasting blood glucose, and glomerular filtration rate were used in analyses, and a description of how these variables were created and categorized, as well as information on missing data, is included in Additional file 1. No blood samples were collected in HUNT 1.

BP measurement procedures carried out during HUNT 2 were identical to those in HUNT 1, with the exception that automated measures were based on oscillometry (Critikon Dinamap 845XT and XL9301; GE Medical Systems Information Technologies, Barrington, IL, USA). SBP, DBP, and heart rate were read three times with 1-minute intervals. In our analyses, the mean value of the second and third readings was used. Mean arterial pressure (MAP) was calculated as one-third SBP + two-thirds of DBP. Pulse pressure (PP) is the difference between SBP and DBP readings.

### Data analyses

Pearson’s χ^2^ tests and independent-samples *t* tests were used for the comparison of categorical and continuous variables between all-cause dementia (*n* = 579) and no dementia (*n* = 24,059). We used logistic regression to examine the association between dementia and BP with SBP, DBP, PP, and MAP as principal predictors entered as continuous variables per 10 mmHg as the measurement unit. A total of four sets of analyses were run separately for both HUNT 1 and HUNT 2 to examine the relationship between BP and all-cause dementia, AD, mixed AD/VaD, and VaD. Stepwise analyses were performed separately, entering SBP, DBP, MAP, and PP alone (model 1); adjusting for age, sex, and education (model 2); entering clinical variables in blockwise fashion (model 3); and finally adding self-reported data (model 4). Interaction effects between sex and age on SBP, DBP, MAP, and PP were examined separately by including interaction terms in fully adjusted models. In separate models, linearity assumptions for continuous BP were examined by introducing polynomial functions and examining predictions in scatterplots. SBP, DBP, MAP, and PP were entered separately as a quadratic measure in fully adjusted models. Age was categorized in 5-year intervals, and results are presented as age entered in models as a continuous variable. All statistical analyses described above were performed using IBM SPSS Statistics version 21 (IBM, Armonk, NY, USA) and Stata version 13 (StataCorp, College Station, TX, USA) software.

## Results

The study population consisted of 24,059 subjects without dementia (52.9% female) and 579 with dementia (66.4% female). Descriptive statistics are shown in Table 1. Persons diagnosed with dementia were older, had a lower education status, and higher percentages were women. The BP averages of SBP, DBP, MAP, and PP in persons with dementia were consistently higher in persons diagnosed with dementia during both HUNT 1 and HUNT 2.

**Table 1:** Characteristics of the study sample

	No dementia	All dementia	Alzheimer disease	Vascular dementia	Alzheimer disease, mixed	*P* value^a^
Total study population, *n*	24,059	579	383	118	78	
Female sex, *n* (%)	12,758 (53.0)	373 (64.4)	257 (67.1)	63 (53.4)	53 (67.9)	0.00
Age in HUNT 1 (1984–1986), years, mean (SD)	50.2 (11.4)	59.9 (7.0)	60.2 (6.8)	58.5 (8.0)	60.7 (6.0)	0.00
HUNT 1^b^: Education completion, *n* (%)						0.00
Primary	14,371 (59.7)	442 (76.3)	292 (76.2)	90 (76.3)	60 (76.9)	
Upper secondary	6032 (25.1)	97 (16.8)	62 (16.2)	22 (18.6)	13 (16.7)	
Higher	3656 (15.2)	40 (6.9)	29 (7.6)	6 (5.1)	5 (6.4)	
HUNT 1: Body mass index, kg/m^2^, mean (SD)	25.4 (3.6)	26.2 (3.8)	26.1 (3.7)	26.6 (4.5)	26.3 (3.2)	0.00
HUNT 1: Pulse, beats/minute, mean (SD)	72.8 (13.0)	73.8 (13.4)	73.6 (14.0)	73.2 (11.8)	75.3 (12.4)	0.07
HUNT 1: Systolic BP, mmHg, mean (SD)	134.3 (20.1)	140.7 (20.0)	140.4 (20.0)	142.1 (20.1)	140.4 (20.2)	0.00
HUNT 1: Diastolic BP, mmHg, mean (SD)	84.5 (10.7)	86.7 (10.7)	86.1 (10.6)	88.1 (10.6)	88.1 (11.3)	0.00
HUNT 1: Mean arterial pressure, mmHg, mean (SD)	101.1 (12.6)	104.7 (12.5)	104.2 (12.4)	106.1 (12.3)	105.5 (13.1)	0.00
HUNT 1: Pulse pressure, mmHg, mean (SD)	49.8 (15.1)	54.0 (15.7)	54.3 (15.7)	54.0 (16.2)	52.4 (14.8)	0.00
Age in HUNT 2 (1995–1997), years, mean (SD)	61.6 (11.4)	71.4 (6.9)	71.6 (6.8)	69.9 (7.9)	72.1 (5.9)	0.00
HUNT 2: Systolic BP, mmHg, mean (SD)	145.4 (22.8)	153.7 (23.2)	152.8 (23.3)	155.4 (21.7)	155.5 (24.4)	0.00
HUNT 2: Diastolic BP, mmHg, mean (SD)	84.1 (12.1)	84.6 (12.8)	83.8 (12.8)	86.9 (11.7)	85.5 (14.1)	0.30
HUNT 2: Mean arterial pressure, mmHg, mean (SD)	104.5 (14.4)	107.6 (14.8)	106.8 (14.9)	110.0 (13.3)	108.8 (16.2)	0.00
HUNT 2: Pulse pressure, mmHg, mean (SD)	61.3 (16.8)	69.0 (17.4)	69.0 (17.3)	68.5 (18.0)	70.0 (17.5)	0.00
HUNT 2: Cholesterol, mmol/L, mean (SD)	6.3 (1.2)	6.7 (1.2)	6.7 (1.2)	6.6 (1.2)	6.6 (1.2)	0.00
HUNT 2: Nonfasting blood glucose, mmol/L, mean (SD)	5.7 (1.7)	5.9 (1.7)	5.8 (1.8)	6.0 (1.8)	5.8 (1.5)	0.03
HUNT 2: Waist-to-hip ratio	0.86 (0.08)	0.85 (0.08)	0.85 (0.08)	0.87 (0.07)	0.85 (0.07)	0.04
HUNT 2: Estimated glomerular filtration rate, ml/minute/1.73 m^2^	70.2 (13.6)	63.1 (12.3)	62.9 (12.3)	64.5 (14.1)	62.2 (9.2)	0.00
Antihypertensive tablet, current or past, *n* (%)	5389 (22.4)	190 (32.8)	120 (31.3)	45 (38.1)	25 (32.1)	0.00
Diabetes mellitus, *n* (%)	307 (1.3)	5 (0.9)	4 (1.0)	1 (3.4)	0	0.38
Myocardial infarction, *n* (%)	314 (1.3)	9 (1.6)	8 (2.1)	1 (0.8)	0	0.60
Angina pectoris, *n* (%)	2047 (8.5)	86 (14.9)	56 (14.6)	18 (15.3)	12 (15.4)	0.00
Stroke, *n* (%)	139 (0.6)	5 (0.9)	1 (0.3)	2 (1.7)	2 (2.9)	0.37
Smoker, *n* (%)						0.00
Never	9933 (41.3)	292 (50.4)	205 (53.5)	45 (38.1)	42 (53.8)	
Ever	9220 (34.2)	197 (34.0)	126 (32.9)	48 (40.7)	23 (29.5)	
Current	5906 (24.5)	90 (15.5)	52 (13.6)	25 (21.2)	13 (16.7)	
Physical activity frequency, *n* (%)						0.00
Never	2361 (9.8)	79 (13.6)	51 (13.3)	20 (16.9)	8 (10.3)	
Less often than once per week	6899 (28.7)	135 (23.3)	82 (21.4)	30 (25.4)	23 (29.5)	
Once per week	6549 (27.2)	135 (23.3)	83 (21.7)	30 (25.4)	22 (28.2)	
Two or three times per week	5506 (22.9)	142 (24.5)	109 (28.5)	19 (16.1)	14 (17.9)	
Nearly every day	2744 (11.4)	88 (15.2)	58 (15.1)	19 (16.1)	11 (14.1)	
Alcohol use during past 2 weeks, *n* (%)						0.00
Abstainer	2447 (10.2)	94 (16.2)	62 (16.2)	19 (16.1)	13 (16.7)	
None	11,014 (45.8)	287 (49.6)	202 (52.7)	52 (44.1)	33 (42.3)	
One to four times	9108 (37.9)	155 (26.8)	91 (23.8)	38 (32.2)	26 (33.3)	
Five to ten times	740 (3.1)	21 (3.6)	14 (3.7)	4 (3.4)	3 (3.8)	
More than ten times	750 (3.1)	22 (3.8)	14 (3.7)	5 (4.2)	3 (3.8)	
Subjective health status						0.00
Poor, *n* (%)	67 (0.3)	3 (0.5)	2 (0.5)	1 (0.8)	0	
Not so good, *n* (%)	3947 (16.4)	144 (24.9)	93 (24.3)	29 (24.6)	22 (28.2)	
Good, *n* (%)	16,053 (66.7)	381 (65.8)	257 (67.1)	74 (62.7)	50 (64.1)	
Very good, *n* (%)	3992 (16.6)	51 (8.8)	31 (8.1)	14 (11.9)	6 (7.7)	

*Abbreviations: BP* Blood pressure, *HUNT 1* Helse Undersøkelse Nord-Trøndelag (1984–1986), *HUNT 2* Helse Undersøkelse Nord-Trøndelag (1995–1997)

^a^ *P* values are derived from *t* tests for continuous variables and χ^2^ tests for the binary variables and indicate differences between “no dementia” and “all dementia”

^b^Model 1: systolic BP

Multiple logistic regression analyses were performed for the total sample and separately for the groups less than and greater than or equal to 60 years of age at the time of HUNT 1 (1984–1986). The results for SBP in the total sample are shown in the upper part of Table 2. Results for DBP, PP, and MAP are presented in Additional file 2. There was a trend for protective effects of high SBP, and this trend tended to be stronger for HUNT 1 than for HUNT 2. In fully adjusted models for age and all other covariates, there was a protective effect of SBP on AD, and a similar, nonsignificant trend was observed for mixed AD/VaD and all-cause dementia. Age, sex, and education (model 2), as well as further blockwise adjustments with the clinical variables (model 3) and general health status (model 4), did not change the estimated effect of SBP much. There was no effect of the quadratic SBP term in fully adjusted models.

**Table 2:** Multiple logistic regression analyses on the association of systolic blood pressure and dementia

	All-cause dementia			Alzheimer disease (AD)			Vascular dementia (VaD)			Mixed AD/VaD		
	OR (95% CI)		NC^a^	OR (95% CI)		NC^a^	OR (95% CI)		NC^a^	OR (95% CI)		NC^a^
(1) Study sample (*n* = 24,638)	HUNT 1	HUNT 2	579	HUNT 1	HUNT 2	383	HUNT 1	HUNT 2	118	HUNT 1	HUNT 2	78
Model 1^b^	**1.16 (1.12–1.20)** ^g^	**1.16 (1.12–1.19)**		**1.15 (1.10–1.20)**	**1.14 (1.10–1.19)**		**1.18 (1.10–1.28)**	**1.19 (1.10–1.27)**		**1.15 (1.04–1.26)**	**1.18 (1.08–1.29)**	
Model 2^c^	**0.94 (0.89–0.99)**	1.03 (0.98–1.09)		0.95 (0.91–1.02)	1.04 (0.98–1.10)		0.96 (0.86–1.07)	1.02 (.92–1.14)		**0.83 (0.72–0.96)**	1.04 (0.92–1.18)	
Model 3^d^	**0.93 (0.88–0.98)**	1.01 (0.96–1.06)		0.94 (0.88–1.01)	1.01 (0.95–1.07)		0.96 (0.85–1.07)	1.01 (.90–1.12)		**0.82 (0.71–0.95)**	1.03 (0.90–1.18)	
Model 4^e^	**0.92 (0.87–0.97)**	1.01 (0.96–1.07)		0.94 (0.88–1.00)	1.01 (0.95–1.07)		0.95 (0.84–1.06)	1.01 (.90–1.13)		**0.82 (0.71–0.95)**	1.04 (0.91–1.18)	
(2) Aged <60 years^f^ (*n* = 18,693)			251			159			60			32
Model 1	**1.21 (1.15–1.28)**	**1.19 (1.13–1.25)**		**1.21 (1.13–1.30)**	**1.19 (1.12–1.27)**		**1.19 (1.01–1.40)**	**1.26 (1.08–1.47)**		0.82 (0.63–1.08)	**1.39 (1.14–1.69)**	
Model 2	1.02 (0.93–1.11)	**1.09 (1.01–1.18)**		1.08 (0.97–1.20)	**1.11 (1.01–1.23)**		1.04 (0.87–1.23)	1.03 (.88–1.22)		**0.68 (0.52–0.90)**	1.07 (0.87–1.33)	
Model 3	1.01 (0.93–1.10)	1.08 (0.99–1.17)		1.08 (0.97–1.20)	**1.11 (1.00–1.22)**		1.03 (0.87–1.23)	1.02 (.86–1.20)		**0.68 (0.52–0.89)**	1.05 (0.85–1.31)	
Model 4	1.01 (0.92–1.10)	1.08 (0.99–1.17)		1.09 (0.97–1.21)	**1.11 (1.00–1.22)**		1.01 (0.85–1.21)	1.01 (.86–1.20)		**0.67 (0.50–0.89)**	1.08 (0.86–1.34)	
(3) Aged >60 years^f^ (*n* = 5945)			328			224			58			46
Model 1	**0.90 (0.84–0.96)**	0.96 (0.90–1.02)		**0.90 (0.83–0.97)**	0.95 (0.88–1.02)		0.92 (0.79–1.06)	0.98 (.85–1.12)		0.91 (0.77–1.07)	0.98 (0.84–1.15)	
Model 2	**0.94 (0.88–0.99)**	0.98 (0.92–1.04)		0.93 (0.86–1.01)	0.97 (0.90–1.05)		0.94 (0.81–1.10)	0.99 (.86–1.14)		0.94 (0.79–1.11)	1.00 (0.85–1.18)	
Model 3	**0.92 (0.86–0.99)**	0.96 (0.90–1.03)		**0.92 (0.84–0.99)**	0.95 (0.88–1.02)		0.94 (0.81–1.09)	0.98 (.85–1.14)		0.94 (0.79–1.11)	1.00 (0.85–1.19)	
Model 4	**0.92 (0.85–0.98)**	0.96 (0.90–1.02)		**0.91 (0.83–0.98)**	0.94 (0.87–1.02)		0.94 (0.81–1.10)	0.99 (.86–1.16)		0.92 (0.77–1.10)	0.99 (0.83–1.17)	

*HUNT 1* Helse Undersøkelse Nord-Trøndelag (1984–1986), *HUNT 2* Helse Undersøkelse Nord-Trøndelag (1995–1997)

^a^Number of dementia cases

^b^Model 1: systolic blood pressure

^c^Model 2: systolic blood pressure, age, sex, education

^d^Model 3: systolic blood pressure, age, sex, education, cholesterol, nonfasting blood glucose, glomerular filtration rate, body mass index, waist-to-hip ratio, pulse

^e^Model 4: systolic blood pressure, age, sex, education, cholesterol, nonfasting blood glucose, glomerular filtration rate, body mass index, waist-to-hip ratio, pulse, history of myocardial infarction, diabetes mellitus, angina, stroke, smoking, subjective health status, physical activity, blood pressure medication, alcohol use

^f^Age when examined in HUNT 1﻿﻿

^g^​p-value <0.05

The bold text represents p-value <=.05

We tested for an interaction effect between sex and SBP and between age and SBP. The age × SBP effect was significant (*p* < 0.01). To further examine this interaction effect, the effect of SBP was examined in different age strata. Splitting the sample into subjects younger than 60 years old at HUNT 1 and 60+ years old at HUNT 1 showed that all the apparent protective effects of high SBP occurred in the oldest group. There was a significant sex × BP interaction effect among subjects younger than 60 years old; however, no sex × BP interaction effect was observed among subjects ≥60 years. The results are shown in the lower part of Table 2. PP had a similar inverse association with the risk of AD (OR 0.99/10-mmHg increase in PP, 95% CI 0.98–0.99), but there were no significant effects of DBP and MAP. These results are shown in Additional file 2: Tables S1, S2, and S3.

To examine whether antihypertensive medication moderated the effect of SBP, samples were split according to antihypertensive use, and results from logistic regression analyses are shown in Table 3. There was an adverse association with AD, PP, and SBP in HUNT 2 in participants <60 years old who reported taking BP medication. Antihypertensive use in participants ≥60 years old did not influence the protective effects of high SBP, because a similar inverse trend was observed among both samples. However, the effect of SBP reached significance only among the participants ≥60 years old who never reported using antihypertensive medication. The BP means in both measurements were highest among participants with a history of taking antihypertensive medication (Table 4).

**Table 3:** Multiple logistic regression analyses and the association of blood pressure and Alzheimer disease, presented according to split age groups (<60, >60 years) and blood pressure medication status (never or ever)

	Never BP medication <60 years old (*n* = 15 267)			Never BP medication >60 years old (*n* = 3 665)			Ever BP medication <60 years old (*n* = 3 329)			Ever BP medication >60 years old (*n* = 2 173)		
	OR (95% CI)		NC^a^	OR (95% CI)		NC^a^	OR (95% CI)		NC^a^	OR (95% CI)		NC^a^
	*HUNT 1*	*HUNT 2*	119	*HUNT 1*	*HUNT 2*	144	*HUNT 1*	*HUNT 2*	41	*HUNT 1*	*HUNT 2*	79
Model 1^b^												
SBP	**1.21 (1.08–1.34)**	**1.17 (1.08–1.27)**		**0.90 (0.82–0.99)**	**0.93 (0.87–0.99)**		**1.19 (1.04–1.35)**	**1.22 (1.06–1.40)**		**0.89 (0.81–0.99)**	0.96 (0.88–1.06)	
DBP	1.08 (0.90–1.30)	0.98 (0.84–1.15)		0.90 (0.76–1.07)	**0.88 (0.77–0.99)**		1.11 (0.84–1.48)	0.87 (0.67–1.12)		0.95 (0.77–1.17)	1.01 (0.86–1.18)	
SBP (with DBP)	**1.28 (1.12–1.46)**	**1.36 (1.22–1.52)**		0.90 (0.81–1.00)	0.96 (0.87–1.06)		**1.25 (1.06–1.47)**	**1.44 (1.22–1.69)**		**0.87 (0.78–0.99)**	0.93 (0.82–1.05)	
DBP (with SBP)	0.85 (0.68–1.06)	**0.67 (0.54–0.82)**		0.99 (0.82–1.22)	0.92 (0.78–1.09)		0.85 (0.60–1.20)	**0.61 (0.45–0.81)**		1.09 (0.86–1.40)	1.09 (0.89–1.33)	
MAP	1.14 (0.96–1.35)	**1.15 (1.02–1.31)**		**0.85 (0.73–0.99)**	**0.90 (0.80–0.99)**		1.20 (0.95–1.51)	1.11 (0.89–1.38)		0.85 (0.71–1.02)	0.98 (0.85–1.13)	
PP	**1.29 (1.12–1.47)**	**1.35 (1.21–1.50)**		**0.88 (0.79–0.98)**	0.93 (0.85–1.03)		**1.26 (1.07–1.48)**	**1.43 (1.22–1.69)**		**0.88 (0.78–0.99)**	0.93 (0.82–1.05)	
Model 2^c^												
SBP	0.98 (0.88–1.10)	0.97 (0.89–1.06)		0.93 (0.84–1.02)	0.94 (0.88–1.01)		1.11 (0.97–1.27)	1.15 (0.99–1.32)		0.92 (0.82–1.02)	0.98 (0.89–1.08)	
DBP	0.86 (0.71–1.04)	0.89 (0.76–1.04)		0.90 (0.75–1.06)	**0.88 (0.77–.99)**		1.06 (0.80–1.41)	0.90 (0.70–1.16)		0.93 (0.75–1.15)	1.00 (0.86–1.18)	
SBP (with DBP)	1.05 (0.92–1.21)	1.04 (.92–1.17)		0.94 (0.84–1.05)	0.98 (0.89–1.08)		1.14 (0.96–1.36)	**1.32 (1.11–1.57)**		0.91 (0.80–1.03)	0.96 (0.85–1.09)	
DBP (with SBP)	0.82 (0.64–1.03)	0.85 (0.69–1.05)		0.95 (0.77–1.16)	0.89 (0.75–1.06)		0.90 (0.63–1.28)	**0.67 (0.50–0.91)**		1.03 (0.80–1.32)	1.05 (0.86–1.29)	
MAP	0.87 (0.73–1.03)	0.96 (0.84–1.09)		0.87 (0.74–1.01)	0.90 (0.81–1.01)		1.10 (0.87–1.39)	1.07 (0.86–1.32)		0.86 (0.72–1.03)	0.99 (0.86–1.14)	
PP	1.05 (0.91–1.20)	1.01 (0.90–1.14)		0.92 (0.82–1.03)	0.95 (0.87–1.05)		1.15 (0.97–1.36)	**1.31 (1.10–1.56)**		0.92 (0.81–1.04)	0.96 (0.84–1.09)	
Model 3^d^												
SBP	0.95 (0.88–1.11)	0.97 (0.89–1.06)		0.92 (0.83–1.01)	**0.92 (0.86–0.99)**		1.11 (0.97–1.28)	1.14 (0.99–1.32)		0.91 (0.82–1.01)	0.98 (0.88–1.08)	
DBP	0.88 (0.72–1.08)	0.89 (0.76–1.05)		0.90 (0.75–1.08)	**0.87 (0.76–0.99)**		1.10 (0.82–1.47)	0.92 (0.71–1.20)		0.91 (0.73–1.13)	1.01 (0.85–1.19)	
SBP (with DBP)	1.05 (0.91–1.20)	1.02 (0.90–1.15)		0.92 (0.82–1.03)	0.95 (0.86–1.05)		1.13 (0.95–1.35)	**1.30 (1.09–1.56)**		0.91 (0.80–1.03)	0.95 (0.84–1.09)	
DBP (with SBP)	0.85 (0.66–1.08)	0.87 (0.70–1.09)		0.98 (0.79–1.20)	0.92 (0.77–1.10)		0.94 (0.66–1.36)	**0.69 (0.50–0.95)**		1.02 (0.79–1.32)	1.06 (0.85–1.32)	
MAP	0.89 (0.74–1.06)	0.96 (0.84–1.10)		0.86 (0.73–1.01)	**0.89 (0.79–0.99)**		1.12 (0.89–1.42)	1.08 (0.87–1.35)		0.85 (0.71–1.02)	0.99 (0.85–1.14)	
PP	1.04 (0.90–1.19)	0.99 (0.88–1.13)		0.89 (0.80–1.00)	0.92 (0.83–1.02)		1.14 (0.96–1.35)	**1.29 (1.07–1.54)**		0.91 (0.81–1.03)	0.95 (0.83–1.08)	
Model 4^e^												
SBP	0.99 (0.88–1.11)	0.97 (0.89–1.06)		**0.90 (0.82–0.99)**	**0.92 (0.85–0.99)**		1.11 (0.96–1.28)	1.14 (0.99–1.32)		0.90 (0.81–1.00)	0.96 (0.87–1.06)	
DBP	0.88 (0.82–1.07)	0.90 (0.76–1.06)		0.89 (0.74–1.06)	**0.86 (0.75–0.98)**		1.11 (0.82–1.51)	0.91 (0.69–1.19)		0.90 (0.72–1.12)	1.00 (0.85–1.19)	
SBP (with DBP)	1.05 (0.92–1.21)	1.02 (0.90–1.15)		0.91 (0.81–1.02)	0.91 (0.76–1.09)		1.12 (0.94–1.35)	**1.30 (1.09–1.56)**		0.90 (0.79–1.02)	0.93 (0.82–1.07)	
DBP (with SBP)	0.83 (0.65–1.07)	0.88 (0.71–1.09)		0.96 (0.78–1.19)	0.68 (0.76–1.09)		0.96 (0.66–1.40)	**0.68 (0.49–0.94)**		1.01 (0.78–1.31)	1.08 (0.86–1.35)	
MAP	0.88 (0.74–1.06)	0.96 (0.84–1.10)		0.84 (0.71–0.99)	**0.88 (0.78–0.99)**		1.12 (0.88–1.43)	1.08 (0.86–1.35)		0.83 (0.69–1.00)	0.97 (0.83–1.13)	
PP	1.05 (0.91–1.21)	1.00 (0.88–1.13)		0.89 (0.79–1.00)	0.91 (0.83–1.01)		1.13 (0.95–1.36)	**1.27 (1.06–1.53)**		0.91 (0.80–1.03)	0.93 (0.81–1.06)	

*Abbreviations: BP* Blood pressure, *DBP* Diastolic blood pressure, *HUNT 1* Helse Undersøkelse Nord-Trøndelag (1984–1986), *HUNT 2* Helse Undersøkelse Nord-Trøndelag (1995–1997), *MAP* Mean arterial pressure, *PP* Pulse pressure, *SBP* Systolic blood pressure

^a^Number of Alzheimer disease cases

^b^Model 1: blood pressure entered alone

^c^Model 2: blood pressure, SBP, DBP, MAP, and PP entered with age, sex, and education

^d^Model 3: SBP, DBP, MAP, and PP entered with, age, sex, education, cholesterol, nonfasting blood glucose, glomerular filtration rate, body mass index, waist-to-hip ratio, and pulse

^e^Model 4: SBP, DBP, MAP, and PP entered with age, sex, education, cholesterol, nonfasting blood glucose, glomerular filtration rate, body mass index, waist-to-hip ratio, pulse, history of myocardial infarction, diabetes mellitus, angina, stroke, smoking, subjective health status, physical activity, blood pressure medication, and alcohol use

The bold text represents p-value <=.05

**Table 4:** Mean systolic and diastolic blood pressure at initial blood pressure (HUNT 1) and follow-up (HUNT 2), according to self-reported antihypertensive status in samples less than and greater than 60 years of age

No dementia				No dementia			
<60 years old				≥60 years old			
Antihypertensive status	Number of subjects	BP HUNT 1, mmHg	BP HUNT 2, mmHg	Antihypertensive status	Number of subjects	BP HUNT 1, mmHg	BP HUNT 2, mmHg
Taking medication (H2)	2651	146.7/93.9	152.8/88.5	Taking medication (H2)	1693	160.2/93.4	163.5/87.2
Previously, not now (H2)	637	139.3/90.4	156.0/91.9	Previously, not now (H2)	409	153.6/90.4	168.9/89.7
Never (H2)	15,148	126.7/81.0	138.8/82.6	Never (H2)	3521	140.7/84.5	155.0/83.6
Alzheimer disease				Alzheimer disease			
<60 years old				≥60 years old			
Antihypertensive status	Number of subjects	BP HUNT 1, mmHg	BP HUNT 2, mmHg	Antihypertensive status	Number of subjects	BP HUNT 1, mmHg	BP HUNT 2, mmHg
Taking medication (H2)	33	153.9/93.5	163.2/85.5	Taking medication (H2)	66	154.4/93.1	159.1/87.3
Previously, not now (H2)	8	147.4/98.0	159.4/91.1	Previously, not now (H2)	13	147.1/86.0	178.6/90.9
Never (H2)	119	131.0/82.0	145.9/82.7	Never (H2)	144	137.6/83.6	150.5/81.7

*Abbreviations: BP* Blood pressure, *H2* HUNT 2, HUNT 1 Helse Undersøkelse Nord-Trøndelag (1984–1986), HUNT 2 Helse Undersøkelse Nord-Trøndelag (1995–1997)

## Discussion

The present study investigated associations between BP measured an average of 17.6 (range 0.6–26.8) years prior to symptom presentation of dementia, AD, and VaD. One of the findings was that high BP did not seem to be a risk factor for dementia when adjusted for age, sex, education, and other covariates in the total sample. Upon further examination, we found that age interactions were apparent in the total sample that required further stratified analyses. Interestingly, in persons 60 years or older, SBP was inversely associated with all-cause dementia, mixed AD/VaD, and AD, but not with VaD. Conversely, among middle-aged subjects (<60 years old), elevated SBP and PP were associated with eventual AD in participants who reported using antihypertensive medication.

The inverse association of SBP with all-cause dementia, mixed AD/VaD, and AD might seem paradoxical because hypertension is generally acknowledged as a risk factor for cognitive decline and dementia [2–4]. However, recent findings suggest that the association between BP and brain health is complex and dependent on factors such as age, chronic hypertension, and antihypertensive medication use [21]. Indeed, inverse associations of BP have been reported in prior studies [11, 12, 22–25].

Our findings raise a question whether high SBP actually has a protective effect against developing AD in a targeted population over 60 years of age. There is evidence to support this because a recent Mendelian randomization study of AD using single-nucleotide polymorphism revealed that higher SBP was associated with lower AD risk [26]. In addition, in studies examining centenarians, researchers have reported that higher BP is associated with higher cognition and functionality [27, 28]. Iadecola et al. summarized the existing evidence that BP in midlife is associated with altered cognitive function in both midlife and late life. However, the association of BP in late life and oldest old age with cognition is less clear, with evidence of both harmful and beneficial effects of high BP on cognition [18, 29]. Although it is known that BP declines in patients with manifest dementia, it is unclear when this decline occurs along the disease trajectory [22]. The Kungsholmen Project indicated that the drop in SBP was evident in subjects over 70 years of age with dementia 3 years prior to onset [4, 30]. The East Boston Cohort also found an inverse association between AD and SBP among participants ≥65 years of age approximately 4 years prior to dementia onset; however, there was no association with BP measured 13 years prior to diagnosis [12]. The East Boston Cohort publication refutes our findings with respect to time lapse prior to disease onset. In our study, the time of onset of dementia was registered on the basis of retrospective data in hospital records and interviews with the closest relatives of nursing home residents; therefore, inaccurate information may have been recorded both because of recall bias and because neurodegenerative disorders are known to begin many years before the patients get symptoms that are very vague in the beginning. However, because measurements in the present study were taken an average of 17.6 (SD 4.6) years prior to diagnosis, we think that it is unlikely that BP decline caused by neurodegeneration can explain these findings. This is in line with a review by Walker et al., who showed limited evidence that mildly elevated BP in late life may be protective against cognitive decline, especially for individuals with a history of long-standing hypertension [21].

In line with previous published studies, our data indicate that higher BP in midlife was associated with greater dementia risk in those who were treated with antihypertensive medication. Midlife BP data have previously been examined in a number of studies providing substantial evidence that there is a longitudinal association between midlife BP and worse cognitive function in later life [31–34]. PP was also found to be associated with greater dementia risk in midlife among participants using antihypertensive medication. PP is a measure of arterial stiffness that increases with age and chronic hypertension, and previous studies showed that elevated PP was associated with cognitive decline and cognitive impairment [35–37]. Collectively, these findings align with well-known midlife studies in which researchers reported adverse associations in hypertensive individuals with a higher prevalence of cardiovascular disease and therefore at increased risk for developing AD [5, 29, 38]. Additional analyses in our study showed that higher initial BP was found among those treated with antihypertensive medication. It is therefore likely that BP in those taking antihypertensive medication was confounded by indication, reflecting that they had higher overall exposure to elevated BP (Table 4).

Researchers in a number of studies have examined whether BP variability over time plays a significant role in the onset and progression of dementia [10, 39, 40]. In particular, SBP variability was found to be associated with significant cognitive deterioration in patients with mild to moderate AD, adding evidence to support the hypothesis that vascular and degenerative processes may interact through an additive or synergistic effect [39, 41]. Although in our present study we did not examine BP variability, the mechanism by which fluctuations in BP and impairment in cerebral blood perfusion may share similar pathologic alterations in cerebral hemodynamics.

It is well known that vascular factors contribute to dementia. The dementia spectrum has previously been described on one end with pure dementia of vascular type, on the other end among those with pure AD, and in between among the largest group with pathologies from both AD and vascular damage [42–45]. On the basis of our data, we found that the association between SBP and VaD was different from the association with AD and may reflect different pathogenesis, as might be expected. Because BP is a known risk factor for stroke, hypertension is often considered a risk factor for VaD [21, 46]. The ICD classification for VaD used in the present study is quite restrictive, and patients who were classified with VaD mostly had a history of stroke or cerebral infarcts that were detected on computed tomographic or magnetic resonance imaging scans, which likely explains our difference in findings.

Our study has a number of strengths, including the very large sample size and the longitudinal design with a long period of follow-up. The dementia diagnoses were validated by experts in the field among a unique, large, population-based sample, and we examined a large set of variables that permitted adjustments and stratification into subgroups. Missing data are hard to avoid when examining longitudinal data, and data analyses were performed using complete-case data. Imputation analyses were not performed, as the data were assumed to be missing at random. Selection bias was probably moderate because the HUNT-HMS study is a prospective, population-based study with a high response rate that provides information about BP measured prospectively up to 27 years prior to onset of dementia. The dementia diagnoses were set by experienced clinicians by using the standardized ICD criteria. However, ascertaining the subtypes of dementia diagnoses was retrospective and based on access to comprehensive medical records from both panels, which were therefore sufficient to subclassify according to the ICD criteria. Furthermore, cognition assessments were not initiated in the county hospitals until 1995, leaving a questionable period during startup in 1995–2000, when scarce numbers of dementia cases were ascertained. A larger fraction of dementia cases were diagnosed during the period 2000–2009, and most cases were identified in 2009–2011. Cognition was not evaluated during HUNT 1 or HUNT 2, but participants were required to complete several questionnaires and underwent extensive screening that intuitively required intact cognition among participants [47]. Although efforts were made to identify participants diagnosed with dementia in the region during 1995–2011 by performing hospital record searches and examining nursing home residents, we had no access to data from individuals with dementia who were under the care of their general practitioner, and these will appear as false-negatives in the data set. However, the proportion of false-negatives to true-negatives in the noncase group is quite low because the prevalence of dementia is, after all, low. Thus, the contamination of the noncase group will not be substantial, and the extent to which the false-negatives affect the observed difference in exposure between the case and noncase groups, and, in turn, the effect estimates, will be little more than inconsequential. Another limitation is an unavoidable characteristic of elderly cohort studies, whereby competing risk from mortality can have affected the results. Finally, the prescription register was established in 2004 in Norway and provides statistics on sales and use from 1990; therefore, data were based on self-reported history, and type of BP-lowering agents was unavailable for analyses [48].

## Conclusions

We conclude that elevated BP does not seem to be a risk factor for dementia when adjusted for age, sex, education, and other covariates. In fact, in persons over 60 years of age, SBP was inversely associated with a prospective dementia diagnosis, whereas in the middle-aged subjects (<60 years old), elevated SBP and PP were associated with eventual AD in participants who reported using BP-lowering medication. These findings are consistent with previously published studies and appear to be dependent on factors such as age, hypertension chronicity, and antihypertensive medication use. Future population-based prospective studies with repeated BP measurements examining BP variability, as well as more detailed data on BP-lowering agents, are needed because the nature of the association between BP and dementia remains unclear.

## Additional files

Additional file 1: Description of baseline data and missing data. (DOCX 16 kb)

Additional file 2: Supplementary Tables 1,2 and 3 present the results on the association of SBP, DBP, MAP, and PPand dementia using multiple logistic regression analyses for the total sample (Table 1); and in participants<60 (Table 2); and >=60 (Table 3).(DOCX 62 kb)

## Abbreviations

AD: Alzheimer disease
BP: Blood pressure
DBP: Diastolic blood pressure
HMS: Health and Memory Study of Nord-Trøndelag (1995–2010)
HUNT 1: Helse Undersøkelse Nord-Trøndelag (1984–1986)
HUNT 2: Helse Undersøkelse Nord-Trøndelag (1995–1997)
ICD-10: International Classification of Diseases, Tenth Revision
MAP: Mean arterial pressure
MCI: Mild cognitive impairment
PP: Pulse pressure
Q1: Questionnaire 1
Q2: Questionnaire 2
SBP: Systolic blood pressure
VaD: Vascular dementia
