# Supplementary material for: Association between blood pressure and Alzheimer disease measured up to 27 years prior to diagnosis: the HUNT Study
Source: Alzheimers Res Ther. 2017 May 31;9:37. doi: 10.1186/s13195-017-0262-x (PMC5452294; doi:10.1186/s13195-017-0262-x)
Supplement: Supplementary file 1 — Description of baseline data and missing data. (DOCX 16 kb) [file 13195_2017_262_MOESM1_ESM.docx]

Additional file 1: description of baseline data and missing data

Baseline data

HUNT 1 stations featured standardized blood pressure measurements for all participants and were managed by trained nurses or technicians using a mercury sphygmomanometer. The blood pressure measurement routinely recorded readings with the participant in a sitting position after having rested a minimum of five minutes. Systolic and diastolic pressure was measured two times, and the second reading was used for our analyses. Heart rate, height, and weight were measured in HUNT 1, following previously described protocol [18]. Sociodemographic data (age, sex, and education) were collected for the HUNT study following standardized protocols [19]. Level of education was categorized, based on “completing primary school”=1, “upper secondary”=2 or “completed at least high school”=3. Respondents were asked to rate how often they drank alcohol over a period of two weeks. Data was categorized as reporting abstaining from alcohol, drinking moderately often (≤ five times during two week), or drinking frequently (> five times during two weeks). Information on physical activity was based on how often (“never”=1 to “almost every day”=5) a participant reported performing physical activity and is used as a categorical variable in models. Subjective general health was measured as a single item with four response categories from “very good”=1 to “bad”=4. History of myocardial infarction (MI), stroke, angina, and diabetes mellitus (DM) were self-reported in HUNT 1 and HUNT 2 and new variables were created combining replies from both HUNT 1 or HUNT2. Waist–hip ratio and body mass index (BMI) were used continuously in analyses. Smoking status was categorized as never, prior, or current smoker from HUNT 2. An item on antihypertensive drugs was dichotomized as “never” or “ever” based on self-reported data from HUNT 1 and HUNT 2. No blood samples were collected in HUNT 1.

BP measurement procedures during HUNT 2 were identical to HUNT 1, with the exception that automated measures were based on oscillometry (Critikon Dinamap 845XT and XL9301, acquired by General Electric (GE) Medical Systems Information Technologies in 2000). Systolic (SBP) and diastolic blood pressure (DBP) and heart rate were read three times with one minute interval. In our analyses mean of second and third reading was used. Mean arterial pressure (MAP) was calculated as: 1/3 systolic BP + 2/3 of diastolic BP. Pulse pressure (PP) is the difference between systolic and diastolic pressure readings. Venous blood was collected during HUNT 2 (1995-1997), and serum total cholesterol was measured by an enzymatic colorimetric cholesterol esterase method. Non-fasting blood glucose was measured by using an enzymatic hexokinase method. Glomerular filtration rate (eGFR) was calculated with the estimated creatinine clearance rate (eCCr) using the Cockcroft-Gault formula.

Missing data

Inclusion criteria are based on both HUNT 1 and 2 survey participation alive in 1995 and who were over the age of 60 in 2011 (n=28,116). The material is presented with complete covariate data (n=24,638) and 3,478 had portions of data missing. The following variables contained missing data: exercise status (n=674); history of taking BP medication (n=78); blood glucose levels (n=60); cholesterol level (n=40); eGFR (n=41); education status (n=990); smoking status (n=627); subjective health status (n=2); waist-hip ratio (n=214); BMI (n=31); pulse (n=106); SBP H1 (n=19); DBP H1 (n=15); SBP H2 (n=81); DBP (n=81); MAP H1 (n=19); MAP H2 (n=82); alcohol (n=777).
