# Supplementary material for: Association between blood pressure and Alzheimer disease measured up to 27 years prior to diagnosis: the HUNT Study
Source: Alzheimers Res Ther. 2017 May 31;9:37. doi: 10.1186/s13195-017-0262-x (PMC5452294; doi:10.1186/s13195-017-0262-x)
Supplement: Supplementary file 2 — Supplementary Tables 1,2 and 3 present the results on the association of SBP, DBP, MAP, and PPand dementia using multiple logistic regression analyses for the total sample (Table 1); and in participants<60 (Table 2); and >=60 (Table 3).(DOCX 62 kb) [file 13195_2017_262_MOESM2_ESM.docx]

|  | |  | | | All-cause dementia | | | | | Alzheimer disease | | | | | | | | | Mixed Alzheimer disease | | | | | | | | | | Vascular dementia | | | | | | | |  |  |  |
| --- | --- | --- | --- | --- | --- | --- | --- | --- | --- | --- | --- | --- | --- | --- | --- | --- | --- | --- | --- | --- | --- | --- | --- | --- | --- | --- | --- | --- | --- | --- | --- | --- | --- | --- | --- | --- | --- | --- | --- |
| **Supplementary Table 1** | | | | | OR (95% CI) | | | | NC^A^ | OR (95% CI) | | | | | | NC | | | OR (95% CI) | | | | | | NC | | | | OR (95% CI) | | | | | NC | | |  |  |  |
| All | |  | | | *HUNT 1* | | *HUNT 2* | | 579 | *HUNT 1* | | | *HUNT 2* | | | 383 | | | *HUNT 1* | | | *HUNT 2* | | | 78 | | | | *HUNT 1* | | | *HUNT 2* | | 118 | | |  |  |  |
| Model 1^B^ | | SBP | | | **1.16 (1.12-1.20)** | | **1.16 (1.12-1.19)** | |  | **1.15 (1.10-1.20)** | | | **1.14 (.110-1.19)** | | |  | | | **1.15 (1.04-1.26)** | | | **1.18 (1.08-1.29)** | | |  | | | | **1.18 (1.10-1.28)** | | | **1.19 (1.10-1.27)** | |  | | |  |  |  |
|  | | DBP | | | **1.21 (1.12-1.30)** | | 1.02 (.96-1.09) | |  | **1.13 (1.03-1.24)** | | | .96 (.89-1.05) | | |  | | | **1.40 (1.16-1.69)** | | | 1.06 (.89-1.26) | | |  | | | | **1.35 (1.16-1.58)** | | | **1.18 (1.03-1.36)** | |  | | |  |  |  |
|  | | SBP (with DBP) | | | **1.15 (1.10-1.21)** | | **1.26 (1.21-1.32)** | |  | **1.18 (1.12-1.25)** | | | **1.27 (1.21-1.34)** | | |  | | | 1.04 (.92-1.19) | | | **1.28 (1.14-1.43)** | | | | | | | **1.13 (1.02-1.25)** | | | **1.22 (1.11-1.34)** | |  | | |  |  |  |
|  | | DBP (with SBP) | | | 1.01 (.92-1.11) | | **.77 (.71-.83)** | |  | .92 (.82-1.03) | | | **.72 (.65-.80)** | | |  | | | **1.32 (1.03-1.71)** | | | **.78 (.63-.98)** | | |  | | | | 1.15 (.94-1.41) | | | .93 (.78-1.11) | |  | | |  |  |  |
|  | | MAP | | | **1.22 (1.15-1.30)** | | **1.16 (1.10-1.22)** | |  | **1.17 (1.09-1.26)** | | | **1.12 (1.04-1.19)** | | |  | | | **1.29 (1.10-1.50)** | | | **1.19 (1.03-1.37)** | | |  | | | | **1.33 (1.18-1.51)** | | | **1.28 (1.14-1.43)** | |  | | |  |  |  |
|  | | PP | | | **1.18 (1.12-1.23)** | | **1.26 (1.21-1.31)** | |  | **1.19 (1.13-1.26)** | | | **1.26 (1.19-1.32)** | | |  | | | 1.11 (.97-1.26) | | | **1.28 (1.15-1.43)** | | |  | | | | **1.17 (1.06-1.30)** | | | **1.24 (1.13-1.36)** | |  | | |  |  |  |
| Model 2^C^ | | SBP | | | .97 (.93-1.02) | | 1.01 (.97-1.05) | |  | .96 (.91-1.01) | | | .99 (.95-1.03) | | |  | | | .95 (.85-1.07) | | | 1.02 (.93-1.13) | | |  | | | | 1.03 (.95-1.13) | | | 1.06 (.98-1.14) | |  | | |  |  |  |
|  | | DBP | | | 1.04 (.97-1.12) | | .98 (.92-1.04) | |  | .97 (.88-1.06) | | | .93 (.86-1.00) | | |  | | | 1.21 (.99-1.48) | | | 1.01 (.85-1.19) | | |  | | | | **1.19 (1.01-1.40)** | | | 1.12 (.98-1.27) | |  | | |  |  |  |
|  | | SBP (with DBP) | | | **.94 (.89-.99)** | | 1.03 (.98-1.09) | |  | .95 (.90-1.02) | | | 1.04 (.98-1.10) | | |  | | | **.83 (.72-.96)** | | | 1.04 (.92-1.18) | | |  | | | | .96 (.86-1.07) | | | 1.02 (.92-1.14) | |  | | |  |  |  |
|  | | DBP (with SBP) | | | **1.12 (1.02-1.23)** | | .94 (.86-1.02) | |  | 1.02 (.91-1.15) | | | **.89 (.80-.99)** | | |  | | | **1.49 (1.16-1.93)** | | | .96 (.77-1.20) | | |  | | | | **1.25 (1.02-1.54)** | | | 1.09 (.91-1.31) | |  | | |  |  |  |
|  | | MAP | | | .98 (.92-1.05) | | 1.01 (.96-1.05) | |  | .93 (.86-1.01) | | | .97 (.91-1.03) | | |  | | | 1.03 (.87-1.23) | | | 1.03 (.89-1.19) | | |  | | | | 1.12 (.98-1.29) | | | **1.13 (1.01-1.27)** | |  | | |  |  |  |
|  | | PP | | | **.94 (.89-.99)** | | 1.02 (.97-1.08) | |  | .95 (.89-1.01) | | | 1.02 (.96-1.08) | | |  | | | **.85 (.73-.99)** | | | 1.00 (.99-1.02) | | |  | | | | .96 (.86-1.08) | | | 1.03 (.93-1.15) | |  | | |  |  |  |
| Model 3^D^ | | SBP | | | .97 (.93-1.01) | | .99 (.96-1.04) | |  | .96 (.91-1.01) | | | .98 (.93-1.02) | | |  | | | .94 (.84-1.06) | | | 1.01 (.92-1.12) | | |  | | | | 1.02 (.93-1.11) | | | 1.05 (.97-1.13) | |  | | |  |  |  |
|  | | DBP | | | 1.05 (.97-1.13) | | .97 (.91-1.03) | |  | .98 (.89-1.08) | | | .92 (.85-1.00) | | |  | | | **1.23 (1.00-1.51)** | | | .99 (.84-1.18) | | |  | | | | 1.16 (.98-1.37) | | | 1.11 (.97-1.27) | |  | | |  |  |  |
|  | | SBP (with DBP) | | | **.93 (.88-.98)** | | 1.01 (.96-1.06) | |  | .94 (.88-1.01) | | | 1.01 (.95-1.07) | | |  | | | **.82 (.71-.95)** | | | 1.03 (.90-1.18) | | |  | | | | .96 (.85-1.07) | | | 1.01 (.90-1.12) | |  | | |  |  |  |
|  | | DBP (with SBP) | | | **1.14 (1.03-1.26)** | | .95 (.87-1.04) | |  | 1.05 (.93-1.18) | | | .91 (.82-1.02) | | |  | | | **1.53 (1.18-1.99)** | | | .96 (.76-1.21) | | |  | | | | **1.25 (1.01-1.54)** | | | 1.10 (.91-1.33) | |  | | |  |  |  |
|  | | MAP | | | .98 (.91-1.04) | | .99 (.94-1.05) | |  | .93 (.86-1.01) | | | .96 (.89-1.02) | | |  | | | 1.03 (.86-1.23) | | | 1.01 (.87-1.17) | | |  | | | | 1.09 (.95-1.26) | | | 1.12 (.99-1.26) | |  | | |  |  |  |
|  | | PP | | | **.92 (.88-.98)** | | 1.00 (.95-1.05) | |  | **.93 (.87-.99)** | | | .99 (.93-1.05) | | |  | | | **.84 (.72-.97)** | | | 1.03 (.91-1.18) | | |  | | | | .95 (.85-1.07) | | | 1.02 (.91-1.14) | |  | | |  |  |  |
| Model 4^E^ | | SBP | | | **.95 (.91-.99)** | | .99 (.95-1.03) | |  | .95 (.90-1.00) | | | .97 (.93-1.02) | | |  | | | .93 (.82-1.05) | | | 1.02 (.92-1.12) | | |  | | | | .99 (.90-1.09) | | | 1.04 (.96-1.13) | |  | | |  |  |  |
|  | | DBP | | | 1.03 (.95-1.12) | | .96 (.90-1.03) | |  | .97 (.87-1.08) | | | **.92 (.84-.99)** | | |  | | | **1.25 (1.00-1.56)** | | | .99 (.83-1.18) | | |  | | | | 1.11 (.93-1.34) | | | 1.09 (.95-1.26) | |  | | |  |  |  |
|  | | SBP (with DBP) | | | **.92 (.87-.97)** | | 1.01 (.96-1.07) | |  | .94 (.88-1.00) | | | 1.01 (.95-1.07) | | |  | | | **.82 (.71-.95)** | | | 1.04 (.91-1.18) | | |  | | | | .95 (.84-1.06) | | | 1.01 (.90-1.13) | |  | | |  |  |  |
|  | | DBP (with SBP) | | | **1.12 (1.01-1.24)** | | .95 (.87-1.04) | |  | 1.03 (.91-1.17) | | | .91 (.81-1.01) | | |  | | | **1.53 (1.17-2.00)** | | | .95 (.76-1.20) | | |  | | | | 1.18 (.95-1.46) | | | 1.08 (.90-1.31) | |  | | |  |  |  |
|  | | MAP | | | .95 (.88-1.03) | | .99 (.93-1.05) | |  | **.91 (.83-.99)** | | | .95 (.89-1.02) | | |  | | | 1.02 (.84-1.24) | | | 1.02 (.87-1.18) | | |  | | | | 1.05 (.90-1.23) | | | 1.11 (.98-1.26) | |  | | |  |  |  |
|  | | PP | | | **.92 (.87-.97)** | | 1.00 (.95-1.05) | |  | **.99 (.98-.99)** | | | **.93 (.87-.99)** | | |  | | | **.83 (.71-.96)** | | | 1.03 (.90-1.18) | | |  | | | | .94 (.83-1.06) | | | 1.01 (.91-1.13) | |  | | |  |  |  |
| Model 4^F^ | | SBP x Age | | | ***.00*** | | ***.00*** | |  | ***.00*** | | | ***.00*** | | |  | | | ***.02*** | | | *.05* | | |  | | | | ***.01*** | | | ***.03*** | |  | | |  |  |  |
|  | | DBP x Age | | | ***.04*** | | *.16* | |  | *.08* | | | *.28* | | |  | | | *.18* | | | *.80* | | |  | | | | *.90* | | | *.29* | |  | | |  |  |  |
|  | | SBP (with DBP) x Age | | | ***.00*** | | ***.00*** | |  | *.08* | | | ***.00*** | | |  | | | *.95* | | | *.05* | | |  | | | | *.89* | | | ***.00*** | |  | | |  |  |  |
|  | | DBP (with SBP) x Age | | | ***.01*** | | ***.00*** | |  | *.09* | | | ***.01*** | | |  | | | *.94* | | | *.91* | | |  | | | | *.94* | | | ***.01*** | |  | | |  |  |  |
|  | | MAP x Age | | | ***.00*** | | ***.00*** | |  | ***.00*** | | | ***.00*** | | |  | | | ***.02*** | | | *.37* | | |  | | | | *.22* | | | *.09* | |  | | |  |  |  |
|  | | PP x Age | | | ***.00*** | | ***.00*** | |  | ***.00*** | | | ***.00*** | | |  | | | *.11* | | | *.00* | | |  | | | | ***.00*** | | | ***.04*** | |  | | |  |  |  |
|  | | SBP x Sex | | | *.12* | | *.18* | |  | *.09* | | | *.13* | | |  | | | *.54* | | | *.76* | | |  | | | | *.99* | | | *.94* | |  | | |  |  |  |
|  | | DBP x Sex | | | *.11* | | *.20* | |  | *.13* | | | *.16* | | |  | | | *.17* | | | *.52* | | |  | | | | *.94* | | | *.99* | |  | | |  |  |  |
|  | | SBP (with DBP) x Sex | | | *.48* | | *.89* | |  | *.35* | | | *.88* | | |  | | | *.25* | | | *.38* | | |  | | | | *.49* | | | *.88* | |  | | |  |  |  |
|  | | DBP (with SBP) x Sex | | | *.92* | | *.78* | |  | *.72* | | | *.75* | | |  | | | *.13* | | | *.30* | | |  | | | | *.49* | | | *.75* | |  | | |  |  |  |
|  | | MAP x Sex | | | *.11* | | *.14* | |  | *.09* | | | *.10* | | |  | | | *.30* | | | *.62* | | |  | | | | *.90* | | | *.96* | |  | | |  |  |  |
|  | | PP x Sex | | | *.06* | | *.22* | |  | *.07* | | | *.15* | | |  | | | *.61* | | | *.76* | | |  | | | | *.67* | | | *.93* | |  | | |  |  |  |
|  | | | |  | | | All-cause dementia | | | | | | Alzheimer disease | | | | | | | | | Mixed Alzheimer disease | | | | | | | Vascular dementia | | | | | | | | | | |
| **Supp Table 2.** | | | |  | | | OR (95% CI) | | | | | NC^A^ | OR (95% CI) | | | | | | NC | | | OR (95% CI) | | | | | NC | | OR (95% CI) | | | | | | | | NC | | |
| >60 | | | |  | | | *HUNT 1* | | *HUNT 2* | | | 328 | *HUNT 1* | | | *HUNT 2* | | | 224 | | | *HUNT 1* | | *HUNT 2* | | | 46 | | *HUNT 1* | | | *HUNT 2* | | | | | 58 | | |
| Model 1^B^ | | | | SBP | | | **.93 (.88-.98)** | | .97 (.92-1.01) | | |  | **.91 (.85-.97)** | | | **.94 (.89-.99)** | | |  | | | .97 (.85-1.11) | | 1.03 (.92-1.16) | | |  | | .98 (.87-1.10) | | | 1.01 (.91-1.12) | | | | |  | | |
|  | | | | DBP | | | .98 (.89-1.09) | | .97 (.90-1.06) | | |  | .92 (.81-1.04) | | | .92 (.84-1.02) | | |  | | | 1.12 (.86-1.45) | | 1.12 (.92-1.36) | | |  | | 1.15 (.91-1.45) | | | 1.07 (.89-1.28) | | | | |  | | |
|  | | | | SBP (with DBP) | | | **.90 (.84-.96)** | | .96 (.90-1.02) | | |  | **.90 (.83-.97)** | | | .95 (.88-1.02) | | |  | | | .91 (.77-1.07) | | .98 (.84-1.15) | | |  | | .92 (.79-1.06) | | | .98 (.85-1.12) | | | | |  | | |
|  | | | | DBP (with SBP) | | | 1.11 (.98-1.26) | | 1.03 (.92-1.14) | | |  | 1.04 (.89-1.21) | | | .98 (.86-1.12) | | |  | | | 1.25 (.91-1.74) | | 1.14 (.88-1.49) | | |  | | 1.27 (.95-1.70) | | | 1.10 (.86-1.40) | | | | |  | | |
|  | | | | MAP | | | **.91 (.83-.99)** | | .97 (.91-1.04) | | |  | **.86 (.78-.96)** | | | .92 (.85-1.00) | | |  | | | .97 (.78-1.21) | | 1.08 (.91-1.29) | | |  | | 1.03 (.85-1.25) | | | 1.08 (.92-1.26) | | | | |  | | |
|  | | | | PP | | | **.89 (.83-.95)** | | .95 (.89-1.01) | | |  | **.88 (.82-.96)** | | | .93 (.86-1.00) | | |  | | | .91 (.77-1.08) | | .98 (.84-1.15) | | |  | | .90 (.77-1.05) | | | .97 (.85-1.12) | | | | |  | | |
| Model 2^C^ | | | | SBP | | | .95 (.90-1.00) | | .98 (.93-1.02) | | |  | **.93 (.87-.99)** | | | .95 (.90-1.01) | | |  | | | .99 (.86-1.13) | | 1.04 (.92-1.17) | | |  | | .99 (.89-1.13) | | | 1.02 (.92-1.14) | | | | |  | | |
|  | | | | DBP | | | .97 (.88-1.08) | | .97 (.90-1.05) | | |  | .91 (.80-1.03) | | | .92 (.83-1.02) | | |  | | | 1.10 (.84-1.43) | | 1.11 (.91-1.36) | | |  | | 1.13 (.89-1.43) | | | 1.07 (.89-1.28) | | | | |  | | |
|  | | | | SBP (with DBP) | | | **.94 (.88-.99)** | | .98 (.92-1.04) | | |  | .93 (.86-1.01) | | | .97 (.90-1.05) | | |  | | | .94 (.79-1.11) | | 1.00 (.85-1.18) | | |  | | .94 (.81-1.10) | | | .99 (.86-1.14) | | | | |  | | |
|  | | | | DBP (with SBP) | | | 1.05 (.92-1.19) | | .99 (.89-1.11) | | |  | .98 (.84-1.15) | | | .95 (.84-1.09) | | |  | | | 1.18 (.85-1.65) | | 1.11 (.85-1.46) | | |  | | 1.21 (.90-1.63) | | | 1.08 (.85-1.38) | | | | |  | | |
|  | | | | MAP | | | .92 (.84-1.00) | | .98 (.91-1.05) | | |  | **.88 (.79-.98)** | | | .93 (.85-1.01) | | |  | | | .98 (.78-1.23) | | 1.09 (.91-1.31) | | |  | | 1.04 (.85-1.26) | | | 1.09 (.92-1.28) | | | | |  | | |
|  | | | | PP | | | **.92 (.86-.99)** | | .97 (.91-1.03) | | |  | **.92 (.85-.99)** | | | .95 (.88-1.03) | | |  | | | .95 (.80-1.13) | | 1.01 (.62-2.87) | | |  | | .92 (.79-1.08) | | | .99 (.86-1.14) | | | | |  | | |
| Model 3^D^ | | | | SBP | | | **.94 (.89-.99)** | | .97 (.92-1.01) | | |  | **.92 (.86-.98)** | | | **.94 (.89-.99)** | | |  | | | .99 (.86-1.14) | | 1.03 (.91-1.17) | | |  | | .99 (.87-1.12) | | | 1.02 (.91-1.13) | | | | |  | | |
|  | | | | DBP | | | .97 (.87-1.08) | | .96 (.88-1.05) | | |  | .90 (.79-1.03) | | | .91 (.82-1.01) | | |  | | | 1.12 (.85-1.46) | | 1.09 (.88-1.35) | | |  | | 1.11 (.87-1.42) | | | 1.07 (.89-1.29) | | | | |  | | |
|  | | | | SBP (with DBP) | | | **.92 (.86-.99)** | | .96 (.90-1.03) | | |  | **.92 (.84-.99)** | | | .95 (.88-1.02) | | |  | | | .94 (.79-1.11) | | 1.00 (.85-1.19) | | |  | | .94 (.81-1.09) | | | .98 (.85-1.14) | | | | |  | | |
|  | | | | DBP (with SBP) | | | 1.06 (.93-1.21) | | 1.01 (.90-1.13) | | |  | .99 (.85-1.17) | | | .97 (.85-1.12) | | |  | | | 1.21 (.86-1.69) | | 1.08 (.81-1.45) | | |  | | 1.23 (.91-1.65) | | | 1.10 (.85-1.41) | | | | |  | | |
|  | | | | MAP | | | **.91 (.83-.99)** | | .96 (.90-1.04) | | |  | **.86 (.77-.96)** | | | .92 (.84-1.00) | | |  | | | .99 (.78-1.24) | | 1.07 (.89-1.29) | | |  | | 1.02 (.83-1.25) | | | 1.09 (.92-1.28) | | | | |  | | |
|  | | | | PP | | | **.91 (.85-.97)** | | .95 (.89-1.01) | | |  | **.90 (.83-.98)** | | | **.92 (.85-.99)** | | |  | | | .95 (.79-1.13) | | 1.01 (.85-1.19) | | |  | | .92 (.78-1.07) | | | .98 (.84-1.14) | | | | |  | | |
| Model 4^E^ | | | | SBP | | | **.93 (.88-.99)** | | .96 (.91-1.01) | | |  | **.90 (.84-.97)** | | | **.93 (.88-.99)** | | |  | | | .98 (.84-1.14) | | 1.02 (.90-1.16) | | |  | | .99 (.87-1.13) | | | 1.03 (.92-1.15) | | | | |  | | |
|  | | | | DBP | | | .97 (.87-1.09) | | .96 (.88-1.05) | | |  | .89 (.78-1.03) | | | .90 (.81-1.01) | | |  | | | 1.13 (.84-1.52) | | 1.08 (.87-1.35) | | |  | | 1.15 (.89-1.49) | | | 1.09 (.89-1.32) | | | | |  | | |
|  | | | | SBP (with DBP) | | | **.92 (.85-.98)** | | .96 (.90-1.02) | | |  | **.91 (.83-.98)** | | | .94 (.87-1.02) | | |  | | | .92 (.77-1.10) | | .99 (.83-1.17) | | |  | | .94 (.81-1.10) | | | .99 (.86-1.16) | | | | |  | | |
|  | | | | DBP (with SBP) | | | 1.06 (.93-1.22) | | 1.01 (.90-1.13) | | |  | .98 (.84-1.16) | | | .97 (.84-1.12) | | |  | | | 1.23 (.87-1.75) | | 1.10 (.82-1.47) | | |  | | 1.23 (.90-1.67) | | | 1.09 (.84-1.41) | | | | |  | | |
|  | | | | MAP | | | **.89 (.81-.99)** | | .96 (.89-1.04) | | |  | **.84 (.74-.94)** | | | **.91 (.83-.99)** | | |  | | | .97 (.75-1.25) | | 1.06 (.88-1.29) | | |  | | 1.04 (.83-1.29) | | | 1.11 (.93-1.31) | | | | |  | | |
|  | | | | PP | | | **.90 (.84-.97)** | | .94 (.88-1.01) | | |  | **.89 (.82-.97)** | | | **.92 (.85-.99)** | | |  | | | .93 (.78-1.12) | | .99 (.83-1.17) | | |  | | .92 (.78-1.08) | | | .99 (.85-1.16) | | | | |  | | |
| Model 4^F^ | | | | SBP x Age | | | *.30* | | *.37* | | |  | *.45* | | | *.52* | | |  | | | *.94* | | *.51* | | |  | | *.39* | | | *.73* | | | | |  | | |
|  | | | | DBP x Age | | | *.73* | | *.08* | | |  | *.88* | | | *.26* | | |  | | | *.99* | | *.22* | | |  | | *.26* | | | *.41* | | | | |  | | |
|  | | | | SBP (with DBP) x Age | | | *.15* | | *.75* | | |  | *.66* | | | *.88* | | |  | | | *.98* | | *.87* | | |  | | ***.02*** | | | *.88* | | | | |  | | |
|  | | | | DBP (with SBP) x Age | | | *.27* | | *.13* | | |  | *.87* | | | *.35* | | |  | | | *.99* | | *.27* | | |  | | *.26* | | | *.35* | | | | |  | | |
|  | | | | MAP x Age | | | *.90* | | *.10* | | |  | *.78* | | | *.20* | | |  | | | *.86* | | *.41* | | |  | | *.71* | | | *.49* | | | | |  | | |
|  | | | | PP x Age | | | *.15* | | *.87* | | |  | *.43* | | | *.91* | | |  | | | *.76* | | *.72* | | |  | | *.10* | | | *.78* | | | | |  | | |
|  | | | | SBP x Sex | | | *.50* | | *.54* | | |  | *.65* | | | *.86* | | |  | | | *.90* | | *.94* | | |  | | ***.02*** | | | *.10* | | | | |  | | |
|  | | | | DBP x Sex | | | *.48* | | *.54* | | |  | *.90* | | | *.88* | | |  | | | *.43* | | *.71* | | |  | | ***.04*** | | | *.15* | | | | |  | | |
|  | | | | SBP (with DBP) x Sex | | | *.94* | | *.69* | | |  | *.16* | | | *.71* | | |  | | | *.08* | | *.21* | | |  | | *.26* | | | *.59* | | | | |  | | |
|  | | | | DBP (with SBP) x Sex | | | *.89* | | *.83* | | |  | *.18* | | | *.73* | | |  | | | *.06* | | *.19* | | |  | | *.74* | | | *.98* | | | | |  | | |
|  | | | | MAP x Sex | | | *.52* | | *.67* | | |  | *.82* | | | *.86* | | |  | | | *.59* | | *.82* | | |  | | ***.02*** | | | *.14* | | | | |  | | |
|  | | | | PP x Sex | | | *.87* | | *.58* | | |  | *.27* | | | *.66* | | |  | | | *.88* | | *.96* | | |  | | ***.02*** | | | ***.04*** | | | | |  | | |
|  | |  | | | All-cause dementia | | | | | | Alzheimer disease | | | | | | | | | | Mixed Alzheimer disease | | | | | | | | | | Vascular dementia | | | | | | | |  |
| **Supp Table 3** | | | | | OR (95% CI) | | | | | NC^A^ | OR (95% CI) | | | | | | | NC | | | OR (95% CI) | | | | | | | NC | | | OR (95% CI) | | | | | NC | | |  |
| <60 | |  | | | *HUNT 1* | | | *HUNT 2* | | 251 | *HUNT 1* | | | | *HUNT 2* | | | 159 | | | *HUNT 1* | | | | *HUNT 2* | | | 32 | | | *HUNT 1* | | | *HUNT 2* | | 60 | | |  |
| Model 1^B^ | | SBP | | | **1.20 (1.13-1.27)** | | | **1.19 (1.13-1.26)** | |  | **1.21 (1.12-1.30)** | | | | **1.19 (1.12-1.28)** | | |  | | | 1.08 (.89-1.30) | | | | 1.13 (.97-1.32) | | |  | | | **1.24 (1.10-1.39)** | | | **1.23 (1.11-1.37)** | |  | | |  |
|  | | DBP | | | **1.24 (1.11-1.39)** | | | 1.03 (.93-1.14) | |  | **1.15 (1.00-1.32)** | | | | .98 (.86-1.12) | | |  | | | 1.49 (1.11-2.00) | | | | .86 (.64-1.16) | | |  | | | **1.37 (1.10-1.70)** | | | **1.36 (1.04-1.53)** | |  | | |  |
|  | | SBP (with DBP) | | | **1.20 (1.10-1.30)** | | | **1.37 (1.27-1.47)** | |  | **1.28 (1.15-1.41)** | | | | **1.40 (1.28-1.53)** | | |  | | | .82 (.63-1.08) | | | | **1.39 (1.14-1.69)** | | |  | | | **1.19 (1.01-1.40)** | | | **1.26 (1.08-1.47)** | |  | | |  |
|  | | DBP (with SBP) | | | 1.00 (.87-1.17) | | | **.70 (.61-.80)** | |  | .86 (.72-1.04) | | | | **.64 (.54-.76)** | | |  | | | **1.87 (1.22-2.85)** | | | | **.58 (.39-.84)** | | |  | | | 1.11 (.82-1.49) | | | .94 (.71-1.24) | |  | | |  |
|  | | MAP | | | **1.26 (1.15-1.39)** | | | **1.19 (1.10-1.30)** | |  | **1.20 (1.07-1.36)** | | | | **1.17 (1.05-1.30)** | | |  | | | **1.31 (1.02-1.69)** | | | | 1.05 (.82-1.34) | | |  | | | **1.39 (1.17-1.67)** | | | **1.33 (1.13-1.56)** | |  | | |  |
|  | | PP | | | **1.30 (1.14-1.35)** | | | **1.36 (1.27-1.46)** | |  | **1.30 (1.17-1.43)** | | | | **1.38 (1.27-1.51)** | | |  | | | .88 (.66-1.18) | | | | **1.37 (1.12-1.66)** | | |  | | | **1.26 (1.07-1.48)** | | | **1.31 (1.13-1.52)** | |  | | |  |
| Model 2^C^ | | SBP | | | 1.01 (.95-1.08) | | | 1.02 (.96-1.08) | |  | 1.01 (.93-1.10) | | | | 1.01 (.94-1.09) | | |  | | | .88 (.72-1.07) | | | | .95 (.81-1.12) | | |  | | | 1.07 (.95-1.22) | | | 1.08 (.96-1.21) | |  | | |  |
|  | | DBP | | | 1.00 (.89-1.12) | | | .94 (.85-1.04) | |  | .92 (.79-1.06) | | | | .89 (.79-1.02) | | |  | | | 1.21 (.89-1.64) | | | | .79 (.59-1.06) | | |  | | | 1.14 (.91-1.43) | | | 1.15 (.95-1.40) | |  | | |  |
|  | | SBP (with DBP) | | | 1.02 (.93-1.11) | | | **1.09 (1.01-1.18)** | |  | 1.08 (.97-1.20) | | | | **1.11 (1.01-1.23)** | | |  | | | **.68 (.52-.90)** | | | | 1.07 (.87-1.33) | | |  | | | 1.04 (.87-1.23) | | | 1.03 (.88-1.22) | |  | | |  |
|  | | DBP (with SBP) | | | .98 (.84-1.15) | | | **.85-.74-.97)** | |  | .84 (.69-1.01) | | | | **.79 (.66-.94)** | | |  | | | **1.85 (1.21-2.85)** | | | | .73 (.49-1.07) | | |  | | | 1.09 (.80-1.48) | | | 1.11 (.84-1.46) | |  | | |  |
|  | | MAP | | | .94 (.90-1.10) | | | 1.01 (.92-1.10) | |  | .94 (.83-1.06) | | | | .98 (.88-1.10) | | |  | | | 1.02 (.78-1.34) | | | | .88 (.68-1.12) | | |  | | | 1.15 (.95-1.39) | | | 1.16 (.98-1.37) | |  | | |  |
|  | | PP | | | 1.02 (.94-1.12) | | | 1.08 (.99-1.17) | |  | 1.07 (.96-1.19) | | | | 1.09 (.99-1.20) | | |  | | | **.71 (.53-.94)** | | | | 1.05 (.85-1.31) | | |  | | | 1.07 (.90-1.27) | | | 1.06 (.90-1.25) | |  | | |  |
| Model 3^D^ | | SBP | | | 1.02 (.95-1.09) | | | 1.02 (.96-1.08) | |  | 1.03 (.95-1.12) | | | | 1.02 (.95-1.10) | | |  | | | .85 (.70-1.05) | | | | .94 (.80-1.13) | | |  | | | 1.05 (.92-1.20) | | | 1.06 (.94-1.20) | |  | | |  |
|  | | DBP | | | 1.02 (.91-1.16) | | | .95 (.85-1.05) | |  | .96 (.83-1.12) | | | | .91 (.80-1.05) | | |  | | | 1.19 (.86-1.64) | | | | .80 (.59-1.08) | | |  | | | 1.10 (.86-1.40) | | | 1.13 (.92-1.39) | |  | | |  |
|  | | SBP (with DBP) | | | 1.01 (.93-1.10) | | | 1.08 (.99-1.17) | |  | 1.08 (.97-1.20) | | | | **1.11 (1.00-1.22)** | | |  | | | **.68 (.52-89)** | | | | 1.05 (.85-1.31) | | |  | | | 1.03 (.87-1.23) | | | 1.02 (.86-1.20) | |  | | |  |
|  | | DBP (with SBP) | | | 1.01 (.86-1.18) | | | **.86 (.75-.99)** | |  | .88 (.72-1.07) | | | | **.81 (.67-.97)** | | |  | | | **1.84 (1.18-2.88)** | | | | .75 (50-1.12) | | |  | | | 1.07 (.78-1.45) | | | 1.11 (.83-1.48) | |  | | |  |
|  | | MAP | | | 1.01 (.91-1.12) | | | 1.01 (.92-1.11) | |  | .98 (.86-1.12) | | | | .99 (.89-1.12) | | |  | | | .99 (.74-1.32) | | | | .88 (.68-1.13) | | |  | | | 1.12 (.91-1.37) | | | 1.13 (.95-1.35) | |  | | |  |
|  | | PP | | | 1.02 (.93-1.11) | | | 1.07 (.98-1.15) | |  | 1.07 (.97-1.19) | | | | 1.08 (.98-1.20) | | |  | | | **.70 (.53-.93)** | | | | 1.04 (.84-1.30) | | |  | | | 1.05 (.88-1.26) | | | 1.04 (.88-1.23) | |  | | |  |
| Model 4^E^ | | SBP | | | 1.01 (.94-1.08) | | | 1.02 (.96-1.08) | |  | 1.04 (.95-1.14) | | | | 1.02 (.95-1.10) | | |  | | | .85 (.68-1.06) | | | | .97 (.82-1.14) | | |  | | | 1.00 (.87-1.16) | | | 1.03 (.91-1.17) | |  | | |  |
|  | | DBP | | | 1.00 (.88-1.14) | | | .93 (.84-1.04) | |  | .96 (.81-1.13) | | | | .91 (.80-1.05) | | |  | | | 1.31 (.91-1.87) | | | | .81 (.59-1.11) | | |  | | | .98 (.75-1.27) | | | 1.07 (.86-1.32) | |  | | |  |
|  | | SBP (with DBP) | | | 1.01 (.92-1.10) | | | 1.08 (.99-1.17) | |  | 1.09 (.97-1.21) | | | | **1.11 (1.00-1.22)** | | |  | | | **.67 (.50-.89)** | | | | 1.08 (.86-1.34) | | |  | | | 1.01 (.85-1.21) | | | 1.01 (.86-1.20) | |  | | |  |
|  | | DBP (with SBP) | | | .99 (.84-1.17) | | | **.85 (.74-.99)** | |  | .88 (.71-1.07) | | | | **.81 (.68-.97)** | | |  | | | **2.00 (1.72-3.87)** | | | | .74 (.49-1.12) | | |  | | | .96 (.70-1.33) | | | 1.05 (.78-1.41) | |  | | |  |
|  | | MAP | | | .99 (.88-1.11) | | | 1.01 (.92-1.10) | |  | .97 (.84-1.12) | | | | 1.00 (.89-1.13) | | |  | | | 1.04 (.75-1.42) | | | | .90 (.69-1.17) | | |  | | | 1.02 (.81-1.28) | | | 1.08 (.90-1.30) | |  | | |  |
|  | | PP | | | 1.02 (.93-1.11) | | | 1.07 (.98-1.16) | |  | 1.08 (.97-1.21) | | | | 1.08 (.98-1.20) | | |  | | | **.68 (.51-.92)** | | | | 1.06 (.85-1.32) | | |  | | | 1.02 (.85-1.22) | | | 1.03 (.87-1.22) | |  | | |  |
| Model 4^F^ | | SBP x Age | | | *.93* | | | *.52* | |  | *.70* | | | | *.42* | | |  | | | *.48* | | | | *.69* | | |  | | | *.30* | | | *.64* | |  | | |  |
|  | | DBP x Age | | | *.73* | | | *.19* | |  | *.66* | | | | *.31* | | |  | | | *.35* | | | | *.87* | | |  | | | *.54* | | | *.57* | |  | | |  |
|  | | SBP (with DBP) x Age | | | *.65* | | | ***.04*** | |  | ***.02*** | | | | ***.04*** | | |  | | | ***.96*** | | | | *.45* | | |  | | | ***.02*** | | | ***.04*** | |  | | |  |
|  | | DBP (with SBP) x Age | | | *.69* | | | ***.02*** | |  | *.57* | | | | ***.04*** | | |  | | | ***.56*** | | | | *.53* | | |  | | | *.56* | | | ***.04*** | |  | | |  |
|  | | MAP x Age | | | *.93* | | | *.82* | |  | *.78* | | | | *.96* | | |  | | | ***.17*** | | | | *.71* | | |  | | | *.14* | | | *.82* | |  | | |  |
|  | | PP x Age | | | *.67* | | | *.11* | |  | *.50* | | | | *.15* | | |  | | | ***.88*** | | | | *.54* | | |  | | | *.58* | | | *.24* | |  | | |  |
|  | | SBP x Sex | | | ***.00*** | | | ***.00*** | |  | ***.02*** | | | | ***.01*** | | |  | | | *.40* | | | | *.55* | | |  | | | ***.02*** | | | *.07* | |  | | |  |
|  | | DBP x Sex | | | ***.00*** | | | ***.01*** | |  | ***.01*** | | | | ***.02*** | | |  | | | *.22* | | | | *.68* | | |  | | | *.09* | | | *.12* | |  | | |  |
|  | | SBP (with DBP) x Sex | | | *.22* | | | *.41* | |  | *.80* | | | | *.98* | | |  | | | *.86* | | | | *.90* | | |  | | | ***.01*** | | | *.20* | |  | | |  |
|  | | DBP (with SBP) x Sex | | | *.82* | | | *.99* | |  | *.31* | | | | *.47* | | |  | | | *.59* | | | | *1.00* | | |  | | | *.05* | | | *.43* | |  | | |  |
|  | | MAP x Sex | | | ***.00*** | | | ***.00*** | |  | ***.01*** | | | | ***.01*** | | |  | | | *.32* | | | | *.57* | | |  | | | *.05* | | | *.11* | |  | | |  |
|  | | PP x Sex | | | ***.00*** | | | ***.01*** | |  | *.06* | | | | ***.04*** | | |  | | | *.33* | | | | *.52* | | |  | | | ***.00*** | | | ***.04*** | |  | | |  |

**Legend.** Results of all blood pressure analyses with SBP, DBP, MAP and PP are provided in supplementary tables 1, 2, and 3. The first table analyses displays multiple logistic regression results of the complete study sample; Supplementary Table 2 in those <60 years of age; and Supplementary Table 3 in those >60 years of age.

^A^Number of dementia cases

^B^ Model 1: Blood pressure measurement (SBP, DBP, MAP, PP) are entered alone in models

^C^Model 2: Blood pressure measurement (SBP, DBP, MAP, PP), age, sex, education

^D^Model 3: Blood pressure measurement (SBP, DBP, MAP, PP), age, sex, education, cholesterol, non-fasting blood glucose, glomerular filtration rate, body mass index, waist hip ratio, pulse

^E^ Model 4: Blood pressure measurement (SBP, DBP, MAP, PP), age, sex, education, cholesterol, non-fasting blood glucose, glomerular filtration rate, body mass index, waist hip ratio, pulse, history of myocardial infarction, diabetes mellitus, angina, stroke, smoking, subjective health status, physical activity, blood pressure medication, alcohol use

^F^Age and sex interaction effects in full model analyses.
